# An Intact Dissecting Baker’s Cyst Mimicking Recurrent Deep Vein Thrombosis

**DOI:** 10.1177/2324709616650703

**Published:** 2016-05-13

**Authors:** Sarah Jamshed, L. Michael Snyder

**Affiliations:** 1 University of Massachusetts Medical School, Worcester, MA, USA

**Keywords:** Baker’s cyst, popliteal cyst, DVT, recurrent DVT, D-dimer

## Abstract

We report a case of a 75-year-old female with a history of acute deep vein thrombosis (DVT) 6 years ago who presented with unilateral calf swelling and pain. D-dimer was normal, and compression ultrasound revealed findings typical of DVT, including an incompressible dilated and hypoechoic peroneal vein. Despite 4 months of anticoagulation for supposed recurrent DVT, pain symptoms persisted and repeat D-dimer and compression ultrasound were unchanged. A magnetic resonance imaging scan to investigate the leg demonstrated a 6-cm dissecting Baker’s cyst extending posterolaterally resulting in venous compression and distal dilation, which appeared to have been confused with a DVT. Ultrasound-guided aspiration of the cyst provided immediate and sustained relief. Herein we provide a review of the literature for the management of this rare scenario.

## Introduction

The differential diagnosis for a unilateral swollen, painful lower extremity includes but is not limited to deep vein thrombosis (DVT), arterial insufficiency, Baker’s (popliteal) cyst rupture, hematoma, lymphedema, thrombophlebitis, thromboangiitis obliterans, venous stasis dermatitis, popliteal aneurysm rupture, neural tumor, malignant histiocytoma, varicose veins, and cellulitis.^1,2^ Most of these diagnoses carry additional identifying features aiding in their recognition, such as characteristic symptoms or skin changes. DVT and ruptured Baker’s cysts are well known to be clinically indistinguishable, and the latter should be kept in mind when managing these patients emergently, as the initial diagnostic approach is the same, but treatment and complications are very different.^3,4^ The initial diagnostic approach consists of a clinical pretest assessment (Well’s score), D-dimer, and compression ultrasonography (CUS) of the leg, if D-dimer is indeterminate. The initial approach is more ambiguous when faced with a recurrent DVT, as no validated algorithm exists as to when to anticoagulate.^5^ Despite potential discrepancies in arriving at the diagnosis, it is paramount that it is the correct one, as the complications of a missed DVT or long-term unnecessary anticoagulative therapy both carry high morbidity. We report a case of an intact complicated Baker’s cyst both clinically and radiologically mimicking a recurrent DVT.

## Case

**Figure 1.**
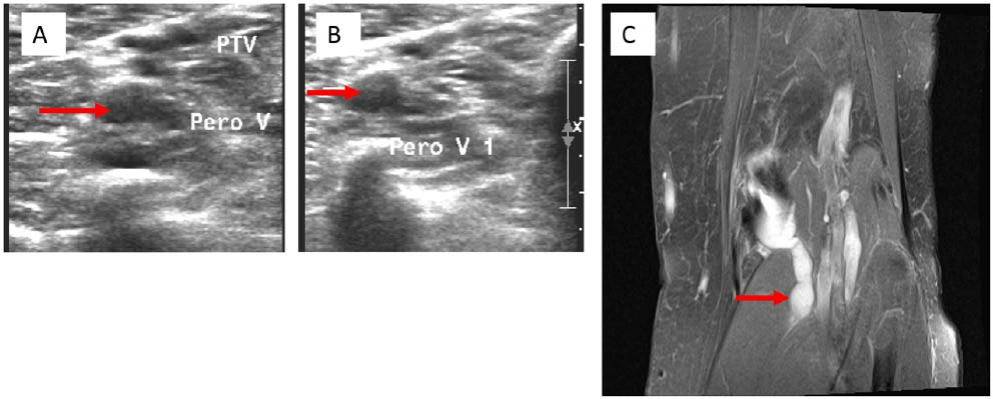
(A) Compression ultrasound (CUS) on initial presentation showing a dilated and echogenic peroneal vessel, consistent with the appearance of a deep vein thrombosis (red arrow). (B) Repeat CUS 4 months later with the same findings, in the absence of clinical symptoms (red arrow). (C) Magnetic resonance angiogram of the left popliteal fossa demonstrating a lobulated 6-cm Baker’s cyst dissecting inferolaterally along the lateral margin of the medial head of the gastrocnemius muscle (red arrow).

A 75-year-old female with a history of chronic lymphocytic leukemia in remission for 23 years and left leg DVT in 2008, on estrogen replacement therapy (ERT) since 1962, presented with several months history of left calf pain. The pain was reproducible on palpation and intermittently accompanied by swelling and erythema. Physical exam demonstrated a 1.5-cm difference in circumference between the 2 calves, which were of equal temperature. Prior medical records indicated a positive D-dimer at 1.21 mg/L and acute thrombus on CUS in 2008, for which she received 6 months of anticoagulative therapy. Based on this and her continued estrogen intake, a recurrent ipsilateral DVT was suspected. A D-dimer assay was well within normal limits at 0.29 mg/L. CUS reported an acute thrombus in 1 of 2 left peroneal veins and an incidental intact 6 cm Baker’s cyst. The supposed thrombus was in the same location as the previous true one in 2008. The patient was taken off ERT and anticoagulated, with plans to continue this regimen indefinitely due to the unprovoked recurrence. Four months into treatment, however, the pain had still not entirely subsided. A repeat CUS interestingly again demonstrated identical findings, with no change in characteristic of the Baker’s cyst. The D-dimer again was within normal range. Magnetic resonance imaging to evaluate the Baker’s cyst revealed extension of the cyst inferolaterally as a lobulated ganglion/synovial cyst dissecting along the lateral margin of the medial head of the gastrocnemius muscle, without evidence of DVT (Figure 1). This was confirmed on magnetic resonance angiogram, demonstrating patent vasculature with no clear-cut filling defect. Heparin and warfarin were discontinued and the patient underwent ultrasound-guided aspiration of the left Baker’s cyst. This yielded 3 mL of straw-colored, blood-tinged, nonpurulent fluid and provided immediate and sustained relief.

## Discussion

This case poses many complications at various stages in diagnosis, which ultimately led to unnecessary treatment. The patient had a history of a true acute DVT in 2008 and resumed ERT once warfarin was discontinued. On repeat presentation with similar but intermittent pain symptoms—though not typical of DVT—is within reason to assume a recurrence, hence an intermediate clinical pretest probability. Though the next step in diagnosis of suspected recurrent DVT is a D-dimer assay, it is at this point not considered a reliable tool and only comes into decision making if CUS is indeterminate. This suggested approach may be due to the fact that D-dimer may remain elevated after the first event, thereby decreasing its dependability in subsequent events.^6^ Suspect recurrent cases are therefore directly triaged for CUS, which remains the gold standard because of cost-effectiveness, wide availability, its noninvasive nature and specificity approaching 100%.^7^ The added confounder in this case is ipsilateral symptomatology, which when confirmed on CUS should ideally have been reported as nondiagnostic, as it is common for previous thrombi to not completely resolve.^8^ In such nondiagnostic cases, the D-dimer is then given weightage. With an intermediate clinical pretest score, negative D-dimer and same vein segment findings on CUS, the recommended approach is serial monitoring with CUS or alternate, more detailed imaging.^9^ This would have not only ruled out a DVT, but would have evaluated the Baker’s cyst in more detail, cited it as the cause of pain, and saved the patient 4 months of discomfort and unnecessary anticoagulation. Anticoagulation would have been warranted had the D-dimer been positive.

Certain factors can play a role in the changing sensitivity and specificity of D-dimer including extent of thrombosis and fibrinolytic activity, duration of symptoms, anticoagulant therapy, comorbidity due to surgical or medical illnesses, inflammatory diseases, cancer, elderly age, pregnancy/puerperium, and, as mentioned before, previous DVT.^10^ However, many previous studies have repeatedly reported the strong negative predictive value of D-dimer (>95%) in patients with low or intermediate clinical pretest,^11-13^ and this highlights the important but adjunct role of D-dimer in the emergency room in the diagnosis of both acute and recurrent DVT.

**Figure 2.**
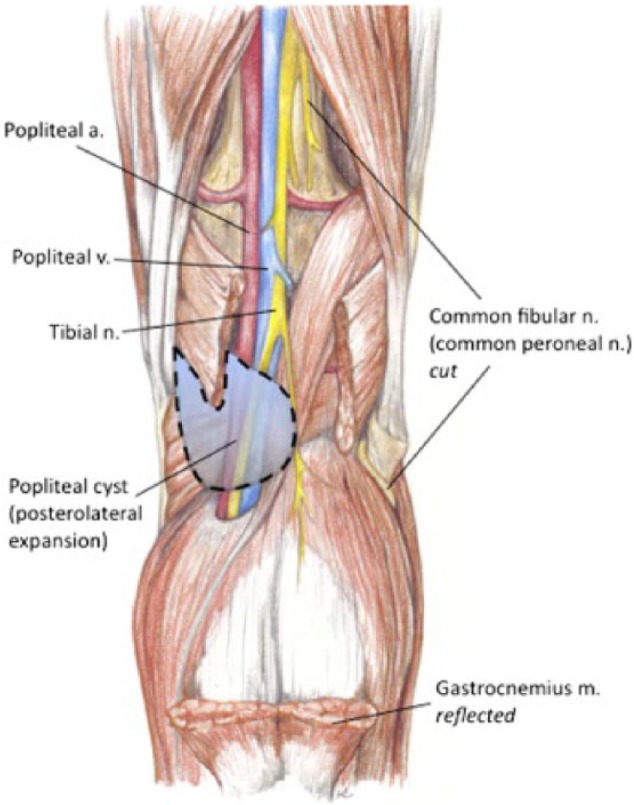
Anatomy of the right popliteal fossa: the dashed line represents a Baker’s cyst following the more unusual course of a posterolateral dissection, thereby causing compression of the neurovascular bundle (highlighted with *dashed line*). In this illustration, the gastrocnemius muscle is reflected for clearer visualization of the cyst, and the deep peroneal nerve is transected.^24^

Popliteal cysts, also known as Baker’s cysts, are enlargements within the popliteal fossa (usually the semimembranosus bursa) filled with synovial fluid. Baker’s cysts are the most common nonvascular condition in this location, but due to their chronic nature can cause various neurovascular manifestations as a result of enlargement and compression. The direction of extension as the cyst develops over time has been found to dictate the subsequent course of disease. Baker’s cysts tend to favor growth in the posteromedial direction due to anatomic lack of support of the synovial capsule in this area.^14^ Accumulation of fluid in this medial location spares the neurovascular bundle. Occasionally the cyst may migrate posterolaterally and cause compression of one of more of the components of the popliteal neurovascular bundle (Figure 2). Symptoms of nerve entrapment manifest as tibial or sciatic neuropathy, resulting in gastrocnemius atrophy.^15-17^ Popliteal vessel compression can cause a thrombophlebitic syndrome^18^ and arterial compression leading to symptoms of claudication of the lower extremity.^19,20^

Few cases of intact posterolateral dissecting Baker’s cysts are reported in the literature. Chong^21^ and Parlov et al^22^ both report this phenomenon and noted the concurrent association of rheumatoid arthritis. Chaudhuri et al also report a similar case of a simultaneous Baker’s cyst and DVT, and also acknowledge that unruptured Baker’s cysts may masquerade radiologically as DVT.^23^ In our case, the absence of DVT was confirmed not only by laboratory testing with D-dimer but also by a repeat false-positive ultrasound scan paired with a negative magnetic resonance angiogram. Ideally, phlebography is the diagnostic test of choice to exclude a calf vein thrombosis when an unruptured Baker’ cyst is identified on ultrasound.^23^ What remains unclear in this patient is how the venous system was selectively affected. It is possible that this is an early result of pressure against an easily compressible structure. In an emergency situation, such complexities are easy to overlook but are important to be aware of.

## Conclusion

No single test is reliable in the diagnosis of recurrent DVT and it is a combination of their results used at the correct time that points toward optimal management. Dissecting Baker’s cysts are a benign entity, but with progressive posterolateral extension can lead to a clinical picture identical to that of DVT. The various complications of Baker’s cysts, including rupture, direct neurovascular compression, and dissection are important to consider when this cyst is identified on initial CUS imaging in a patient with unilateral calf pain and swelling and negative D-dimer.
